# The role of non-standard translation in *Candida albicans* pathogenesis

**DOI:** 10.1093/femsyr/foab032

**Published:** 2021-05-22

**Authors:** Ana Rita Bezerra, Carla Oliveira, Inês Correia, Ana Rita Guimarães, Gonçalo Sousa, Maria João Carvalho, Gabriela Moura, Manuel A S Santos

**Affiliations:** Department of Medical Sciences, Institute of Biomedicine – iBiMED, University of Aveiro, 3810-193 Aveiro, Portugal; Department of Medical Sciences, Institute of Biomedicine – iBiMED, University of Aveiro, 3810-193 Aveiro, Portugal; Department of Medical Sciences, Institute of Biomedicine – iBiMED, University of Aveiro, 3810-193 Aveiro, Portugal; Department of Medical Sciences, Institute of Biomedicine – iBiMED, University of Aveiro, 3810-193 Aveiro, Portugal; Department of Medical Sciences, Institute of Biomedicine – iBiMED, University of Aveiro, 3810-193 Aveiro, Portugal; Department of Medical Sciences, Institute of Biomedicine – iBiMED, University of Aveiro, 3810-193 Aveiro, Portugal; Department of Medical Sciences, Institute of Biomedicine – iBiMED, University of Aveiro, 3810-193 Aveiro, Portugal; Department of Medical Sciences, Institute of Biomedicine – iBiMED, University of Aveiro, 3810-193 Aveiro, Portugal

**Keywords:** *Candida albicans*, non-standard translation, pathogenesis, drug resistance, genetic diversity, evolution

## Abstract

*Candida albicans* typically resides in the human gastrointestinal tract and mucosal membranes as a commensal organism. To adapt and cope with the host immune system, it has evolved a variety of mechanisms of adaptation such as stress-induced mutagenesis and epigenetic regulation. Niche-specific patterns of gene expression also allow the fungus to fine-tune its response to specific microenvironments in the host and switch from harmless commensal to invasive pathogen. Proteome plasticity produced by CUG ambiguity, on the other hand is emerging as a new layer of complexity in *C. albicans* adaptation, pathogenesis, and drug resistance. Such proteome plasticity is the result of a genetic code alteration where the leucine CUG codon is translated mainly as serine (97%), but maintains some level of leucine (3%) assignment. In this review, we dissect the link between *C. albicans* non-standard CUG translation, proteome plasticity, host adaptation and pathogenesis. We discuss published work showing how this pathogen uses the fidelity of protein synthesis to spawn novel virulence traits.

## Teaser

*Candida albicans* uses the fidelity of protein synthesis to spawn novel virulence traits.

## INTRODUCTION

The genetic code establishes the rules that govern the transfer of genetic information from nucleic acids to proteins. By establishing a unambiguous correspondence between codons and amino acids, the genetic code allows for stable inheritance of phenotypic variation produced by proteins upon which natural selection acts (Crick 1968). Despite this, numerous deviations to the standard genetic code have been discovered in both prokaryotes and eukaryotes (Knight, Freeland and Landweber 2001a; Koonin and Novozhilov 2009; Keeling 2016). Most of these alterations occurred in mitochondria due to their small genomes and independent translation machinery—ribosomes and tRNAs (Knight, Landweber and Yarus 2001b; Ling, OʼDonoghue and Soll 2015). Although alterations in nuclear genomes are less common, they include sense and nonsense codon reassignments, as well as codon unassignments (Miranda, Silva and Santos 2006; Kollmar and Muhlhausen 2017), with sense-to-sense codon reassignments having the highest potential to boost proteome plasticity.

The only reported cases of sense-to-sense codon reassignment in nuclear genomes invariably involves the leucine CUG codon. The identity of this codon changed three times during the evolution of budding yeasts, with two clades translating the CUG codon as serine (CUG-Ser), one clade translating it as alanine (CUG-Ala) and two clades translating it canonically as leucine (CUG-Leu) (Krassowski *et al*. 2018). Among the CUG-Ala species are *Pachysolen* and *Nakazawaea*, which are mostly used in biotechnological applications (Muhlhausen *et al*. 2016; Riley *et al*. 2016). Most species with biomedical relevance belong to the CTG-clade that comprise the most pathogenic *Candida* species (Fitzpatrick *et al*. 2006; Butler *et al*. 2009). Within this clade, *C. cylindracea* translates CUGs as serine only, while *C. guilliermondii*, *C. dubliniensis*, *C. zeylanoides*, *C. tropicalis* and *C. albicans* ambiguously decode the CUG codon as serine and leucine (Tuite and Santos 1996; M. A. Santos *et al*. 1997; Suzuki, Ueda and Watanabe 1997; M. A. Santos *et al*. 2011). Another example of dual codon identity occurs in the *Ascoidea*-clade species, where *Ascoidea asiatica* translates CUG stochastically as serine or leucine. In this species, residues encoded by CUG codons in key structural sites are strictly avoided (Muhlhausen *et al*. 2018).

Unlike *A. asiatica*, CUG-encoded residues may exist at functionally relevant positions in *C. albicans* and other CTG-clade *spp*. Most residues are located at the protein surface, where both leucine and serine can be incorporated without major impact on protein structure or function, but a few are located in positions where a serine or other polar amino acid is conserved in homologous proteins (Rocha *et al*. 2011). This is particularly intriguing because inaccurate production of proteins is generally viewed as a nuisance to biological systems (Kapur and Ackerman 2018; M. Santos *et al*. 2018). Nonetheless, recent data challenged these assumptions and strengthen the idea that protein mistranslation is a double-edged sword with potentially adaptive functions (Ribas de Pouplana *et al*. 2014; Schwartz and Pan 2017). Several studies show that, under stress, increased dual-translation of a codon enhances microbial fitness and modulates host-microbe interactions (Carlson *et al*. 2009; Netzer *et al*. 2009; Evans *et al*. 2018).

The concept of adaptive translation is greatly exemplified in *C. albicans*, where natural dual-translation of CUGs (which are normally translated as ∼3% Leu and ∼97% Ser but can change) expands the proteome exponentially (6438 CUG-containing genes can potentially encode 283 billion proteins, under a 50%–50% scenario) (Gomes *et al*. 2007; Rocha *et al*. 2011; M. A. Santos *et al*. 2011). The biological implications of this are profound, as each cell may contain a unique combination of protein molecules, generating an enormous biological complexity in this important human fungal pathogen. In this review, we dissect how protein diversity caused by variable translation of the CUG codon is emerging as a new key player in *C. albicans* adaptation, pathogenesis and drug resistance.

## NON-STANDARD TRANSLATION IN *CANDIDA SPP*

**Figure 1.**
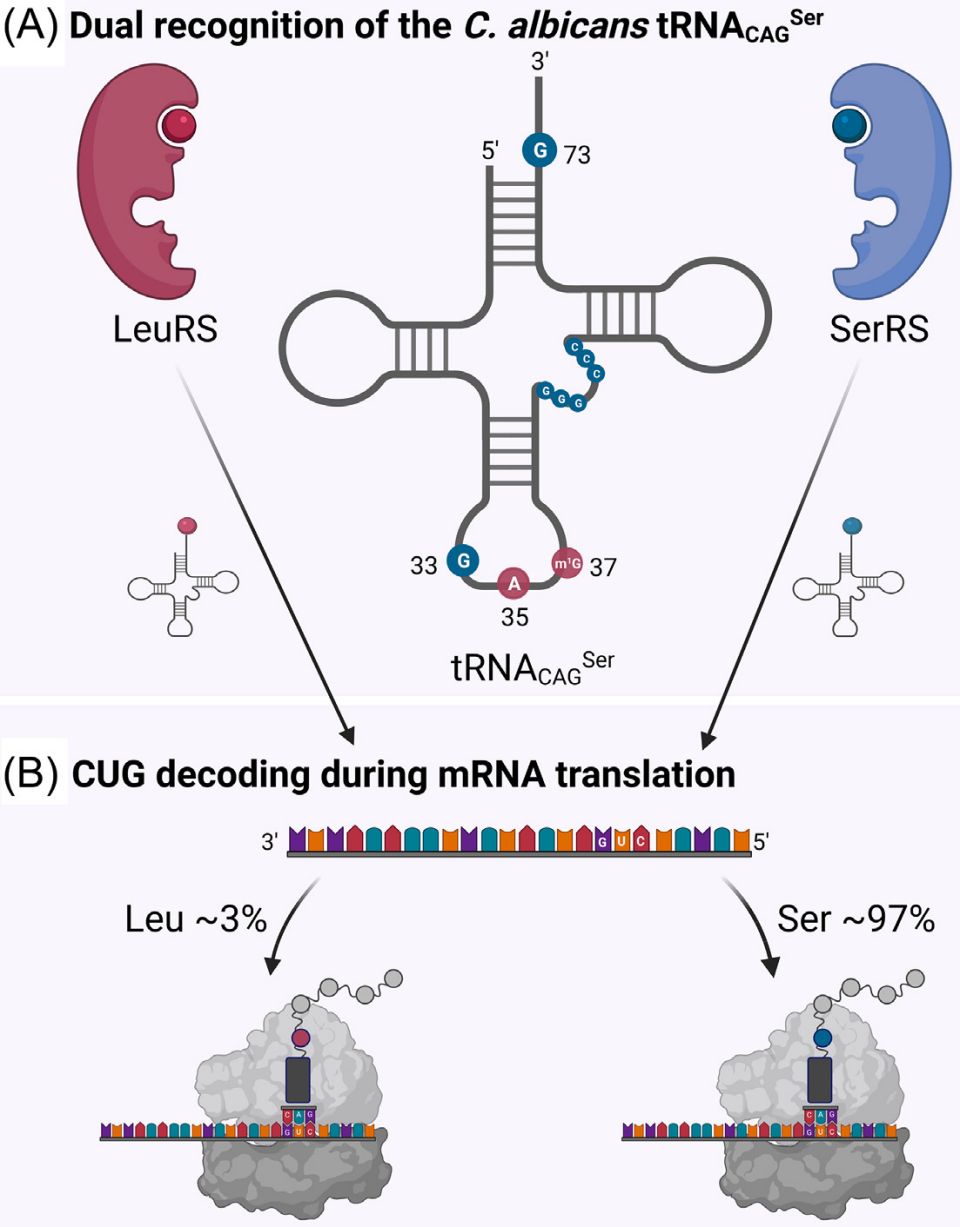
Schematic illustration of the C. albicans tRNA_CAG_^Ser^. **(A) Dual recognition of the C. albicans tRNA_CAG_^Ser^_._**The C. albicans tRNA_CAG_^Ser^ contains identity elements for both LeuRS and SerRS. The LeuRS recognizes A_35_ and m^1^G_37_ in the anticodon loop (red circles). The SerRS recognizes the discriminator base (G_73_) and three GC base pairs of the extra-arm (blue circles). Notably, the presence of G_33_, instead of U_33_, in the anticodon U-turn decreases leucylation efficiency of the tRNA_CAG_^Ser^ in vitro (Miranda, Silva and Santos 2006) This double recognition by LeuRS and SerRS leads to the synthesis of two aminoacyl-tRNAs (charged with Ser or Leu) that compete for CUG codons. **(B) CUG decoding during mRNA translation**. Under standard growth conditions, C. albicans translates approximately 97% of CUG codons as Ser and approximately 3% as Leu (Miranda, Silva and Santos 2006; Gomes *et al*. 2007). Figure created with Biorender.com.

At least 31 *Candida spp*. are known to cause disease in humans. Although the incidence of *Candida spp*. varies geographically, globally it is estimated that ∼92% of the infections are caused by only five species: *C. albicans, C. glabrata, C. tropicalis, C. parapsilosis* and *C. krusei* (*Pichia kudriavzevii*) (Pfaller *et al*. 2010; Guinea 2014; Gabaldon *et al*. 2016). *C. albicans* is the most common pathogen isolated, but the prevalence of non-*albicans* species has been increasing in the past years. Other increasingly important causes of candidiasis include: *C. lusitaniae, C. guilliermondii, C. dubliniensis, C. kefyr (Kluyveromyces marxianus), C. famata* (*Debaryomyces hansenii*)*, C. incospicua, C. rugosa, C. dubliniensis, C. norvegensis* (*Pichia norvegensis*) and the multi-drug resistant *C. auris* (Pfaller *et al*. 2010; Papon *et al*. 2013; Gabaldon *et al*. 2016; CDC 2019). Apart from *C. glabrata, C. krusei*, *C. kefyr*, *C. norvegensis*, and *C. incospicua*, all *Candida spp*. listed belong to the CTG clade, a large group of yeasts that reassigned the leucine CUG codon (Butler *et al*. 2009; M. A. Santos *et al*. 2011; Papon *et al*. 2013; Du *et al*. 2020). Within this clade, *C. albicans, C. zeylanoides, C. lusitaniae, C. tropicalis, C. parapsilosis, C. guilliermondii and C. rugosa* naturally translate the leucine CUG codons as both serine and leucine (Suzuki, Ueda and Watanabe 1997; Gomes *et al*. 2007; M. A. Santos *et al*. 2011). This group of *Candida spp*. with dual CUG codon translation comprises most of the pathogenic *Candida* species, whose change of identity of the CUG codon is mediated by a novel tRNA_CAG_^Ser^ that contains identity elements for both the seryl- and leucyl-tRNA synthetases (SerRS and LeuRS, respectively). SerRS recognizes the 3 GC base pairs in the extra arm and the discriminator base (G73), while LeuRS recognizes the A35 and m^1^G37 in the anticodon loop of the tRNA molecule. This leads to double recognition by SerRS and LeuRS and to synthesis of two different aminoacyl-tRNAs, specifically a Ser-tRNA_CAG_^Ser^ and a Leu-tRNA_CAG_^Ser^, that compete for CUG codons at the ribosome A-site during mRNA translation (Fig. 1). The mischarged Leu-tRNA_CAG_^Ser^ is not edited by the LeuRS nor discriminated by the translation elongation factor 1 (eEF1A) and both Leu and Ser are incorporated into the proteome at CUG positions (Santos, Perreau and Tuite 1996; M. A. Santos *et al*. 1997; Gomes *et al*. 2007).

The evolutionary pathway of the CUG codon reassignment has been thoroughly discussed (Schultz and Yarus 1996; M. A. Santos *et al*. 2004; Miranda, Silva and Santos 2006; Moura, Paredes and Santos 2010), and is not in the scope of this review. However, these studies were the first to shed light on the mechanisms underlying the tolerance to codon ambiguity. Early reports noted that CUG is a rare codon in CTG clade species (Brown *et al*. 1991; Sugiyama *et al*. 1995; Suzuki, Ueda and Watanabe 1997) and tends to appear in non-housekeeping genes (Suzuki, Ueda and Watanabe 1997). Indeed, codon usage analysis revealed that the CUG codon is repressed in *C. albicans* (unlike *C. cylindracea*, the CUG 100% Ser decoder, which uses CUG as the preferred Ser codon) and CUG codons appear at serine codon sites in *S. cerevisiae* proteins rather than in Leu CUG codon positions (Massey *et al*. 2003; Gomes *et al*. 2007; Butler *et al*. 2009). This indicates that CUG reassignment and ambiguous decoding erased most of the CUG codons in the CTG clade ancestor and that new CUG sites arose in Ser codon positions (Massey *et al*. 2003; M. A. Santos *et al*. 2011).

Gomes and colleagues demonstrated that CUG is ambiguously decoded *in vivo* in *C. albicans* by quantifying Leu and Ser levels at CUG sites (Gomes *et al*. 2007), using a direct protein mass spectrometry (MS) analysis. The levels of leucine incorporation at CUG sites in *C. albicans* grown in standard growth conditions were 2.96% (Gomes *et al*. 2007). In the same study, recombinant *C. albicans* cells tolerated incorporation of 28% of Leu at CUG sites without significant impact on fitness. It was calculated that 13 074 CUG codons were distributed over more than a half (66%) of *C. albicans* genes. Considering that each gene has on average three CUGs, an ambiguity level of 2.96% could generate 6.7 × 10^6^ novel proteins per cell in standard growth conditions. For this reason, it was considered that *C. albicans* proteome has a statistical nature (Gomes *et al*. 2007).

To evaluate the impact of CUG codon ambiguity on the proteome, Rocha and colleagues conducted a deep structural analysis of proteins containing CUG-encoded residues (Rocha *et al*. 2011). The alignment of 680 protein sequences with CUG-encoded residues in *C. albicans* with orthologs of six other fungal species revealed that 90% of CUG codons are located at non-conserved positions. The same pattern was observed for the other CTG clade species analysed, whereas CUGs in *S. cerevisiae* are evenly distributed in the protein sequences (Rocha *et al*. 2011). The distribution pattern of CUGs in the CTG clade species suggested that reintroduction of CUGs in the genomes, after the CUG reassignment, was selected to avoid protein misfolding caused by serine substitution for leucine. Proteins with CUG-encoded residues were uniformly distributed across diverse functional categories. However, the 10% of conserved CUG-encoded residues were mainly located in proteins involved in biological processes associated with virulence and pathogenesis, such as biofilm formation, mating, morphogenesis and adhesion. They also correlated with signal transduction, suggesting a pivotal role for CUG decoding ambiguity in pathogen–host interaction (Rocha *et al*. 2011).

To validate the functional impact of Ser-for-Leu substitutions, Rocha and colleagues determined the crystal structures of the two isoforms of SerRS (SerRS_Ser197 and SerRS_Leu197) (Rocha *et al*. 2011). SerRS has a CUG-encoded serine residue at position 197 located at the dimer interface. The Ser-for-Leu substitution induces a local rearrangement, which increases slightly (27%) the activity of the SerRS_Leu197 isoform (Rocha *et al*. 2011; Robbins, Caplan and Cowen 2017). Similarly, Zhou and co-workers showed that the unique CUG-encoded residue present at position 919 in LeuRS is not conserved among eukaryotes, but is involved in tRNA binding and aminoacylation. Ser-for-Leu substitution at the unique CUG site increased ∼30% the LeuRS activity without causing major structural alterations (Zhou *et al*. 2013). These studies suggest that leucine incorporation at CUG sites could be regulated by a fine balance of the SerRS and LeuRS isoforms activity. However, further investigation is needed to clarify this issue as we are still far from understanding the molecular mechanisms that regulate CUG codon ambiguity.

Another example of the impact of CUG codon ambiguity on protein function is the *C. albicans* eukaryotic translation initiation factor (eIF)4E. This cap-binding protein mediates mRNA binding to the ribosome and to translation initiation factors (Hinnebusch and Lorsch 2012). Feketová and colleagues showed that eIF4E has two variants due to CUG ambiguous decoding: eIF4E_Leu116 and eIF4E_Ser116 (Feketova *et al*. 2010). This residue is located in the surface of the protein, adjacent to the eIF4G binding region. Ser-for-Leu did not impair activity, but the eIF4E_Leu116 was temperature sensitive (Feketova *et al*. 2010), suggesting that *C. albicans* may reprogramme translation from CAP-dependent to CAP-independent mRNAs at high temperatures.

In another study, Leu-CUG translation decreased the stability and activity of the protein kinase Cek1 without major structural alterations. Cek1 contains a single CUG-encoded residue at a conserved position and is a key kinase of the MAPK cascade directly linked to morphogenesis in *C. albicans*. Incorporation of Ser at this CUG site induced the autophosphorylation of the conserved tyrosine residue of the Cek1 ^231^TEY^233^ motif and increased its intrinsic kinase activity *in vitro*. Unlike the Ser-Cek1 variant, the Leu-Cek1 variant is not autophosphorylated at the ^231^TEY^233^ motif within the kinase activation loop (Fraga *et al*. 2019). Therefore, these *in vitro* studies demonstrate that Leu/Ser-CUG isoforms of *C. albicans* proteins can be functional despite some differences in activity and/or specificity. It is also possible that CUG ambiguous decoding could affect protein-protein interactions, disrupting existing networks or producing new ones.

As the CTG clade shows some variability in reassignments and *C. albicans* tolerates high level of ambiguity at CUG sites (Gomes *et al*. 2007), the question if *C. albicans* tolerates ambiguity at other codons arose. To answer this, Simões and colleagues tested if *C. albicans* tolerates codon ambiguity at other codons (Simoes *et al*. 2016). For this, *C. albicans* strains were engineered to express tRNA_UGA_^Ser^ genes with mutated anticodons. Several mutant tRNAs that were able to read codons encoding amino acids with different chemical properties, namely Leu, Ala, Gly, Lys, Thr and Tyr were engineered. This approach relied on the fact that eukaryotic seryl-tRNA synthetase (SerRS) does not recognize the tRNA Ser anticodon loop (Lenhard *et al*. 1999), so alterations in the anticodon do not affect tRNA serylation. In other words, each targeted codon was decoded by the mutant tRNA_UGA_^Ser^ and by its cognate tRNA. It is important to note that the codons targeted in this study follow a similar codon usage as CUG. Interestingly, expression of the recombinant tRNAs had little impact on fitness (Simoes *et al*. 2016).

The high tolerance of *C. albicans* to codon ambiguity, alongside the functional consequences of Ser/Leu decoding mentioned above, raise the question of the overall implications of such ambiguity to *Candida spp*. biology. Recent studies provide evidence that CUG ambiguity may indeed be relevant for adaptation. The double identity of the CUG codon creates statistical proteins whose implications in the many facets of *C. albicans* virulence will be dissected in the following sections.

## PHENOTYPIC AND GENOMIC IMPACT OF CUG AMBIGUITY

Quantification of Leu incorporation at CUG sites using an MS-based reporter system revealed that wild-type *C. albicans* cells can increase CUG codon ambiguity up to ∼5%, when grown in low pH conditions (Gomes *et al*. 2007). Although small, an increase of incorporation of Leu at CUG sites from the basal 3% to 5% could generate an additional 4.2 × 10^6^ statistical polypeptides (Gomes *et al*. 2007). Remarkably, recombinant *C. albicans* strains encoding a yeast Leu tRNA_CAG_^Leu^, and incorporating 28% Leu at CUG sites displayed very high morphologic diversity, including ovoid opaque cells, pseudo-hyphal and hyphal morphologies. The phenotypes observed in highly ambiguous cells were similar to the phenotypes that are induced by environmental signals, such as high temperature, low pH and serum. Expression of a mutant *S. cerevisiae* Leu-tRNA_CAG_ in *C. albicans* strains increased Leu incorporation at CUG sites and exposed hidden phenotypic variability, including the same morphological changes reported by Gomes and colleagues, but also enhanced activity of extracellular hydrolases (aspartic proteinases and phospholipases). High-level CUG codon ambiguity also induced mating by upregulating the key regulator of white-opaque switching, which was reflected on the increase of mating competent opaque cells. Furthermore, it revealed up-regulated expression of genes involved in cell adhesion and hyphal development (Miranda *et al*. 2007). This is in line with other data showing that CUG-encoded residues are enriched at conserved positions in proteins associated with biofilm formation, morphological switching, and adhesion (Rocha *et al*. 2011).

Miranda and co-workers also showed that *C. albicans* cells that incorporated 28% Leu at CUG sites had higher adherence to fibronectin and gelatin (Miranda *et al*. 2013). This is relevant because *C. albicans* is a successful commensal of mammalian hosts due to its capacity to adhere to host constituents and to avoid immune clearance. The authors proposed two mechanisms to explain the high adherence phenotype. First, knowing that most CUG-encoded residues are located on the surface of proteins and that membrane and cell wall genes are particularly enriched in CUG codons (Rocha *et al*. 2011), CUG mistranslation may increase cell surface hydrophobicity due to increased substitution of polar Ser for the non-polar Leu. Second, presence of Leu at the two CUG-encoded residues (positions 379 and 433) in the adhesin Als3 altered its function and increased cell flocculation and substrate adhesion. Moreover, high level of Leu-CUG translation produced variability in the cell wall proteome that altered the cell surface exposure of 1,3-β-glucan and reduced phagocytosis by macrophages, compared to wild-type cells (Miranda *et al*. 2013). Together, these results indicate that proteome expansion generated by codon ambiguity can be a mechanism to generate cell surface variability and possibly to contribute to antigenic variation to avoid recognition by the immune system, which is particularly relevant in the fungal host-interaction.

To test the limits of CUG-Leu decoding and its role in virulence, a set of recombinant *C. albicans* strains incorporating between ∼20% and ∼98% of Leu at CUG sites were constructed (Bezerra *et al*. 2013). Although high-level Leu-CUG translating strains displayed slower growth in rich medium, when grown with oxidative agents or antifungal drugs, particularly azoles, recombinant strains grew better than the wild-type strain (Bezerra *et al*. 2013). Similarly, Simões and coworkers reported that *C. albicans* strains that misincorporate Ser at other codons generally had increased growth rate when exposed to oxidative and osmotic stress (Simoes *et al*. 2016). Interestingly, the strain that incorporates ∼98% of Leu at CUG sites was resistant to SDS and CuSO_4_. Considering that SDS limits cell growth in yeasts by disrupting cell membranes and cell walls (Heilmann *et al*. 2013; Reyna-Beltrán *et al*. 2019; Schroeder and Ikui 2019), and copper toxicity is used by the innate immune system to kill fungal cells by causing membrane damage (Douglas and Konopka 2019), these findings indicate once again that high levels of Leu incorporation at CUG sites may induce extensive cell wall remodelling. Furthermore, experiments where high CUG mistranslating strains were exposed to human monocyte-derived dendritic cells showed increased in vitro release of the inflammatory ILs IL-1β, IL-10, and IL-12p70 cytokines, supporting that CUG ambiguity also modulates the immune responses to the fungus. To assess the pathogenic potential of high Leu-CUG translating strains *in vivo*, C57BL/6 mice were inoculated intragastrically. Although fungal load was higher in mice infected with the wild-type strain, colonization by Leu misincorporating strains was associated with a clear inflammatory pathology in the stomach of infected mice, as revealed by the increased mucin content of goblet cells and the number of infiltrating cells. Moreover, levels of the proinflammatory cytokines TNFα and IL-17A were higher, and the anti-inflammatory IL-10 levels were lower relative to the wild-type strain. These results suggested that a diversified inflammatory response is elicited by dynamic Leu misincorporation at CUGs (Bezerra *et al*. 2013).

Genome analysis of high-level Leu-CUG translating and wild-type strains showed that increasing levels of CUG ambiguity have a profound impact on the genome (Bezerra *et al*. 2013). Indeed, high levels of CUG-Leu translation induced loss of heterozygosity (LOH) in a 300-kb region of the chromosome V in strains that incorporate ∼80% and ∼98% of Leu and for the entire chromosome R in the strain incorporating ∼80% of Leu at CUG sites. The observed LOH events affected mainly genes with unknown function, but it also affected genes involved in regulation of biological processes, organelle organization and stress response. Sequence analysis also indicated that the number of SNPs increased proportionally to the level of Leu-CUG translation. The strain with ∼98% of Leu incorporation at CUG sites, for example, accumulated higher number of unique SNPs and a higher number of SNPs was found in genes enriched with CUG codons. SNPs accumulated mainly in genes with unknown function, followed by genes associated with filamentous growth, cell adhesion, metabolic processes, and transcription regulation. Thus, the genomic changes were found to be correlated with some of the phenotypes observed in these recombinant cells (Bezerra *et al*. 2013). In another genomic study, experimental evolution of strains misincorporating Ser at other codons showed a similar great impact on the genome. Genome analysis showed stronger accumulation of LOH events and SNPs in cells with higher codon ambiguity than in control cells (Simoes *et al*. 2016). Interestingly, a SNP in the *JAB1* gene was found in all highly ambiguous strains; a gene involved in protein degradation by neddylation (Sela, Atir-Lande and Kornitzer 2012; Simoes *et al*. 2016). Taken together, it shows that codon ambiguity can lead to genome and phenotypic diversification with high adaptation potential, namely in the evasion of immune system and in azole tolerance.

## CUG AMBIGUITY ACCELERATES ACQUISITION OF DRUG RESISTANCE

The increasing impact of *Candida* species on human health is mostly due to the existence of a limited number of approved antifungal drugs and the emergence of drug resistance. Currently, there are only five classes of antifungal agents: polyenes (amphotericin B), azoles (fluconazole, itraconazole, posaconazole, voriconazole and isavuconazole), echinocandins (caspofungin, micafungin and anidulafungin), allylamines (terbinafine) and antimetabolites (flucytosine). The scarcity of antifungals is partially explained by the conservation of eukaryotic gene functions between fungal pathogens and the human host (Perlin, Rautemaa-Richardson and Alastruey-Izquierdo 2017; Robbins, Caplan and Cowen 2017; Fisher *et al*. 2018), leading to a reduced number of viable safe targets. Of all classes, the most used against *Candida* infections belong to the azoles, mainly fluconazole, due to its great efficacy, low toxicity, bioavailability and low cost. However, the extensive use of fluconazole has resulted in increased resistance to azoles, turning therapies increasingly ineffective (Castanheira *et al*. 2017; Robbins, Caplan and Cowen 2017). Therefore, understanding the molecular mechanisms of drug resistance is of crucial importance for the medical community.

Several studies have identified two main molecular mechanisms of acquisition of azole resistance in *C. albicans*. First, overexpression of the drug target gene *ERG11* (14a-lanosterol demethylase) reduces the direct impact of the drug (Oliver *et al*. 2007). Second, increased efflux of the drug from cells by ABC transporters (encoded by *CDR1* and *CDR2*) (Coste *et al*. 2006) or by the major facilitator superfamily efflux pump (encoded by *MDR1*) (Dunkel *et al*. 2008) reduces the effective intracellular drug concentration. In both cases, such alterations can result from point mutations in genes encoding those proteins, in transcription factors regulating mRNA expression levels (Coste *et al*. 2006; Dunkel *et al*. 2008), or from increased copy number of the relevant genes, via genome rearrangements (Selmecki, Forche and Berman 2006).

The importance of epigenetic pathways in mediating drug resistance in fungi is also well established and involve tuning of a pre-programmed response without permanent genetic changes (Chang *et al*. 2019). Regulation of translational fidelity may act similarly, as it affords the cell the opportunity of producing an entirely novel set of proteins from the same genetic background. Although most of these proteins will likely be neutral or deleterious in function, a small subset may acquire novel functions entirely. This is the case in bacteria, where Javid *et al*. showed that sequence variation of antibiotic targets results in resistant phenotypes (Javid *et al*. 2014). In this study, wild-type mycobacteria and strains with high and low translational fidelity were compared to investigate a direct role of mistranslation in antibiotic tolerance. Low translational fidelity strains had increased antibiotic tolerance both *in vivo* and *in vitro*. When the antibiotic target was purified from low and high mistranslating strains, the mistranslated protein from the high mistranslating strains had increased resistance. This corroborated that protein variation was responsible for the observed phenotype (Javid *et al*. 2014).

Although mistranslation is associated with antibiotic resistance in bacteria (Balashov and Humayun 2002; Amar, Al Mamun and Humayun 2009; Javid *et al*. 2014), this link is far less explored in fungal pathogens. As discussed in the previous sections of this review, in yeast and fungi, codon ambiguity leads to genome and phenotypic diversification and increases adaptation potential, including azole tolerance (Gomes *et al*. 2007; Miranda *et al*. 2007; Paredes *et al*. 2012; Bezerra *et al*. 2013; Miranda *et al*. 2013; Kalapis *et al*. 2015). In *C. albicans*, Weil and colleagues investigated how CUG non-standard translation contributes to the acquisition of antifungal resistance by evolving high-level Leu-CUG translating and wild-type strains in the absence and presence of fluconazole and comparing their resistance trajectories during evolution (Weil *et al*. 2017). Results showed that increased levels of mistranslation lead to increased tolerance and accelerate the emergence of fluconazole resistance. Genome sequencing, array-based comparative genome analysis, and transcriptional profiling revealed that during evolution in fluconazole, the range of mutational and gene deregulation variations were distinctively different and broader in strains that incorporate Leu at high level. While the expression of genes of the ergosterol biosynthesis pathway, encoding the molecular target of fluconazole, was upregulated in both wild-type and high mistranslating resistant strains, the drug efflux pathway was affected differently (Weil *et al*. 2017). The wild-type strain acquired a known gain-of-function mutation in the transcription factor *MRR1*, a positive transcriptional regulator of the multidrug efflux pump gene *MDR1* (Morschhauser *et al*. 2007; Dunkel *et al*. 2008). On the other hand, the high-level Leu-CUG translating strain acquired a previously described A736V gain-of-function mutation in *TAC1*, the transcriptional activator of the *CDR1* and *CDR2* genes, which encode the ABC transporters (Coste *et al*. 2006; Lohberger, Coste and Sanglard 2014). Taken together, and although the stochastic nature of mutations should be stressed here, these results indicated that both strains activated the traditional mechanisms of azole resistance, nonetheless through different drug efflux pathways. Also, the evolution of the hypermistranslating strain, in both the absence and presence of fluconazole, resulted in the fast accumulation of LOH events. A large LOH region in chromosome 5 (Chr5) appeared exclusively in the presence of fluconazole, which is consistent with previous studies of fluconazole-resistant *C. albicans* isolates (Selmecki, Forche and Berman 2006; Ford *et al*. 2015). Interestingly, analysis of the relative synonymous codon usage in genes affected by LOH events during evolution revealed three key aspects: (i) the number of CUG codons differed between alleles (a and b) of genes that lost heterozygosity; (ii) genes with a higher number of CUG codons in allele a than allele b (a > b) underwent a reduction in CUGs by changing them to other codons; (iii) genes with a lower number of CUGs in allele a than allele b (a < b) had an increase in the number of CUG codons by changing other codons to CUG. The same pattern was observed in copy number variation analysis of the evolved high-level Leu-CUG translating strain. This resistant strain showed loss of chromosomal repeat regions in Chr2, Chr4 and Chr6, and increased copies of Chr1, Chr4, Chr5 and ChrR. Within these amplified chromosomal regions were a large proportion of genes involved in both translation and drug resistance. Therefore allele-specific gene functions may contribute to rapid adaptation to fluconazole and CUG codon usage and translation seems to have a balancing effect on such genomic events (Bezerra *et al*. 2013; Kalapis *et al*. 2015; Weil *et al*. 2017).

## CONCLUSION

**Figure 2.**
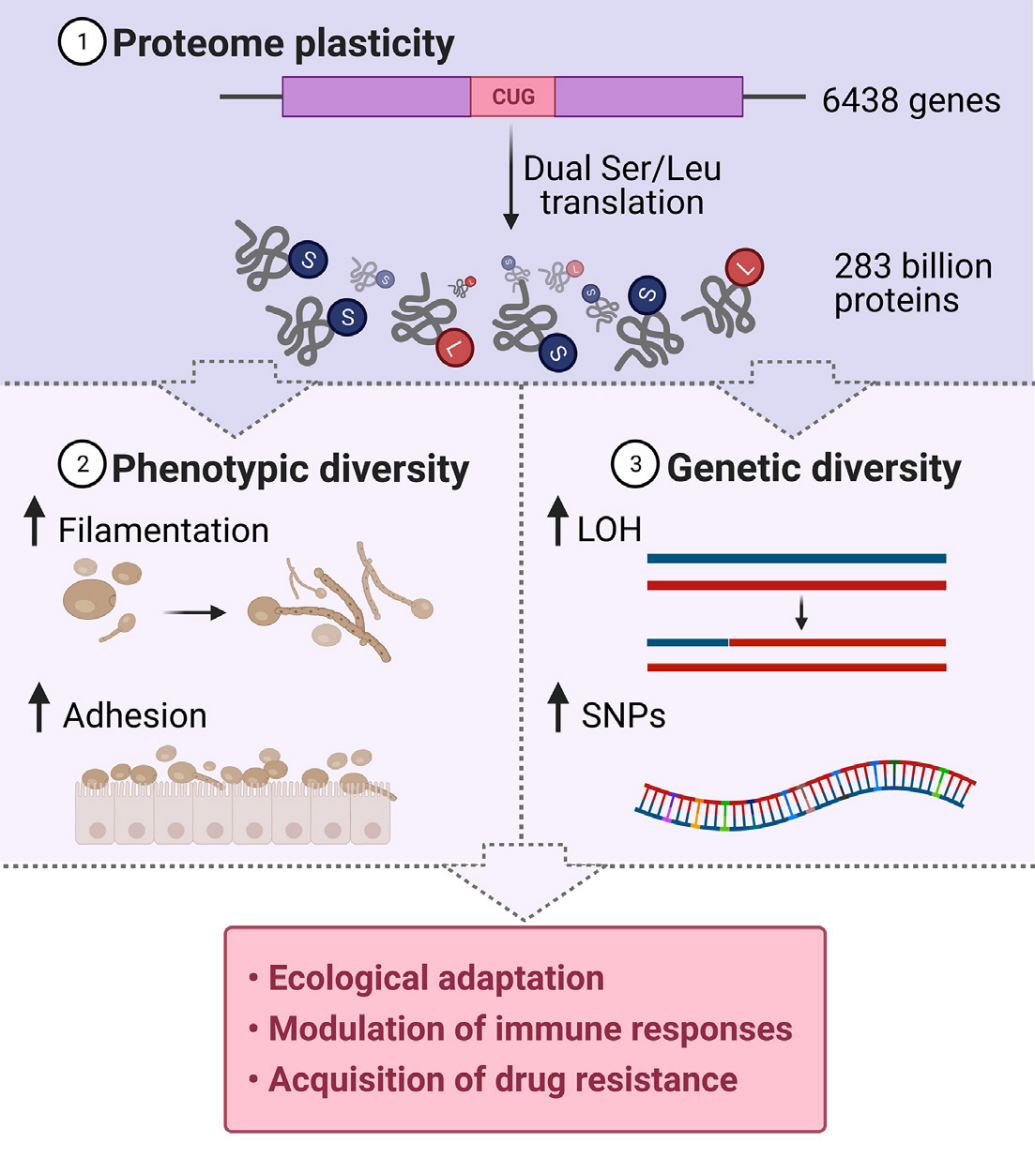
Diagram representing the impact of CUG codon ambiguity in *C. albicans* biology. To date, it has been demonstrated three main consequences of Ser/Leu dual translation. (**1**) **Proteome plasticity:** it is estimated that the 6438 *C. albicans* genes containing CUGs generate 283 billion different proteins (Gomes *et al*. 2007). The impact of the proteome expansion was shown to generate (**2**) **Phenotypic diversity**, with high Leu-CUG translating cells show increased filamentation and adhesion to host substrates (Miranda *et al*. 2007; Miranda *et al*. 2013). CUG codon ambiguity also induces (**3**) **Genetic diversity**. *C. albicans* cells with high Leu-CUG levels displayed a higher number of LOH events and accumulation of SNPs into their genomes. Although, *C. albicans* signalling pathways directly affected by CUG codon ambiguity are still largely unexplored, the phenotypes uncovered support the hypothesis that CUG ambiguity modulates immune responses and accelerates ecological adaptation and acquisition of drug resistance (Bezerra *et al*. 2013; Weil *et al*. 2017). Figure created with Biorender.com.

The effect of codon ambiguity on phenotypic and genetic diversity of *Candida* spp. adds a new dimension to the study of genome evolution, phenotypic variation, ecological adaptation, drug resistance and virulence in humans. The direct impact of CUG ambiguity in *C. albicans* signalling pathways is still largely unexplored, but the accumulating evidence on phenotypes uncovered support the hypothesis that CUG ambiguity modulates host-pathogen interaction and immune responses and accelerates the evolution of drug resistance (Fig. 2). The implications are wide and should be further explored at the molecular level.
